# Missing Diagnosis, Pain, and Loss of Function in Older Adults with Rheumatoid Arthritis and Insufficiency Fractures: A Qualitative Study of the Patient’s Perspective

**DOI:** 10.3390/geriatrics5040094

**Published:** 2020-11-17

**Authors:** Pia Simonsen Lentz, Anna Havelund Rasmussen, Aysun Yurtsever, Dorte Melgaard

**Affiliations:** 1 Department of Physiotherapy and Occupational Therapy North Denmark Regional Hospital, Bispensgade 37, DK-9800 Hjoerring, Denmark; psl@rn.dk (P.S.L.); annahavelundrasmussen@gmail.com (A.H.R.); 2 Department of Rheumatology, North Denmark Regional Hospital, Bispensgade 37, DK-9800 Hjørring, Denmark; ayyu@rn.dk; 3 Centre for Clinical Research, North Denmark Regional Hospital, DK-9800 Hjoerring, Denmark; 4 Department of Clinical Medicine, Aalborg University, DK-9000 Aalborg, Denmark

**Keywords:** rheumatoid arthritis, insufficiency fracture, stress fracture, rehabilitation, pain, delayed diagnosis, qualitative research

## Abstract

Rheumatoid arthritis (RA) is characterised by a chronic, progressive inflammation in the joints and leads to substantial pain, disability, and other morbidities. Few studies document the occurrence of insufficiency fractures, but no studies document the patient’s perspective on incurring an insufficiency fracture. The aim of this qualitative study was to explore the patients’ perspective on how insufficiency fractures influence their level of activity and to detect their need for rehabilitation. Two focus-group interviews were performed with 10 patients diagnosed with RA and insufficiency fractures. The data from the focus-group interviews were subjected to thematic analysis to provide a sense of the important themes. The 10 patients were all females, aged 57–88 years. Magnetic resonance imaging were performed at a mean of six months and seven days. All patients identified the delayed diagnosis of fracture as a significant burden. They experienced pain but did not receive a diagnosis. When the patients were immobilised, some of them were offered aids such as crutches, which they were unable to use due to their RA. The patients needed a focus on diagnosis and individually customised rehabilitation, taking into account RA and including guidance concerning daily activities, aids, and the regain of physical function.

## 1. Introduction

Rheumatoid arthritis (RA) is the most common form of inflammatory arthritis [1], and the disease is characterised by chronic, progressive, systematic inflammation. This inflammation leads to substantial pain, disability, and other morbidities [2]. Patients with RA have an increased risk of osteoporosis and osteoporotic fractures [3], and other studies document that women with RA have an increased risk of fractures compared to women without RA [4,5,6,7,8]. Osteoporotic fracture is associated with the risk of falling [9,10]. An increased risk of osteoporosis and osteoporotic fractures is well reported, and the higher risk is related to inactivity or corticosteroid therapy [11].

Only occasionally has the medical literature reported the occurrence of insufficiency extremity fractures in rheumatoid arthritis [12,13]. An insufficiency fracture occurs when the mechanical strength of a bone is reduced to the point that physiological stress, which would not fracture a healthy bone, breaks a weak one. The condition that causes reduced bone strength typically does so throughout the skeleton (e.g., osteoporosis, osteomalacia, or osteogenesis imperfecta) but may be more localised (e.g., demineralisation in a limb due to disuse) [14].

Rheumatologists in the North Denmark Regional Hospital noticed some cases in which new-onset knee or ankle pain was misinterpreted as arthritis activity by the clinicians but was later shown to be insufficiency fractures [15]. X-ray examination did not show the insufficiency fractures, but MRI examination did reveal them. The fractures are at risk of being overlooked, and this fact may lead to a delay in diagnosis and the risk of ineffective treatment with, for example, steroid injections [12,13,15,16]. The included patients’ RA status was well known, and they often experienced symptoms such as pain, reduced functionality, and loss of quality of life (QOL). The insufficiency fractures were treated conservatively, and the patients were immobilised for several weeks [15]. Due to their RA, the patients had difficulty using crutches and wheelchairs, and they often became care-dependent [17].

The complexity of the course of diseases due to RA and the immobilisation of the patients while the bone heals makes it relevant to involve the patient’s experiences and perspective. This qualitative study aimed to explore what patients diagnosed with RA and insufficiency fractures experienced relevant during and after immobilisation.

## 2. Materials and Methods

### 2.1. Study Design

Two focus-group interviews were conducted to gather information about rehabilitation needs in patients with RA and insufficiency fractures during and after immobilisation. A loose model was used to structure the moderator guide with five starting questions in order to create an exploratory data production [18].

- “Did you experience pain?—if yes, how did the pain influence your life?”
- “How did you manage your daily activities?”
- “Were you challenged with physical activity when you were immobilized?”
- “Was your mood, sleep or social life influenced by the time you had the fracture?”
- “Is there anything you find important to tell about the time you had the fracture?”

This method was furthermore chosen to open the possibility of interaction among the group members and allow them to explore and clarify individual and common perspectives [19].

### 2.2. Participants

The rheumatologists identified a total of 15 patients with insufficiency fractures in the period from 2010 to 2015. They were all women aged 57–89. They had an average RA disease duration of 14.2 years, and all patients had a diagnosis delay of insufficiency fracture from about one month to more than one year and nine months. Two patients died, and the remaining 13 patients were invited for the interview, but three declined the invitation. A drop out analysis was not constructed.

The study was conducted in the physio- and occupational therapy unit in North Denmark Regional Hospital, Hjørring, in 2015. All the participating patients in this study were affiliated with the rheumatology department, North Denmark Regional Hospital. Initially, the patients were sent a letter to inform them about the aim of the study, and later they were contacted by telephone. Ten patients, aged 57–88 years and fulfilling the classification criteria for RA and insufficiency fractures, participated. The participants had an average RA disease duration of 14.2 years, and a magnetic resonance imaging (MRI) had been performed at a mean of six months and seven days after pain onset, as illustrated in Table 1.

The participating patients were divided into two focus groups. Each of the two groups was constructed based on age ranges, 57–69 years and 71–88 years. The groups were based on age due to the presumption that the groups then would be more homogenous to level of function and similar experiences. The purpose was furthermore to make a clear sense of the participants’ reactions during the discussions and observe similarities and differences in their opinions and experiences [19].

### 2.3. Data Collection and Analysis

The focus-group interviews were carried out in an informal clinical setting, each lasting between 45 min and one hour. The interviews were conducted by P.S.L. and A.H.R. as moderators, one moderator for each group. If the patients responded individually to the moderator’s questions, they were encouraged to talk and interact with each other [20]. The moderator guide consisted of questions of the following wide issues concerning difficulties in performing activities of daily living, difficulties in physical activity and exercise, modified function level, and delay of diagnosis.

Both focus-group interviews were audiotaped and afterwards transcribed by P.S.L. All patients were initially given a pseudonym from the numbers 1–10, later used in the processing of patients’ statements. The analysis was conducted by coding in which meaningfully related data items were assigned to a unique theme [18,19].

The thematic analysis of the focus groups’ data provided a sense of important themes. The authors discussed and studied in detail the statements of different patients, leading to the identification of key candidate themes. Subthemes and overarching themes were developed further and refined by discussion between the authors. Finally, the main themes were agreed upon and named.

### 2.4. Ethical Considerations

According to Danish legislation, the registration and publication of data from clinical registries do not require patient consent or approval by ethics committees, but all patients nevertheless signed informed written consent to ensure a high level of information. The Danish Data Protection Agency approved the study (No. 2015-41-4158).

## 3. Results

### 3.1. The Thematic Analysis Identified Two Main Themes: Delayed Diagnosis and Loss of Functionality Delayed Diagnosis

All patients identified the missing diagnosis as a significant burden. They were feeling an unknown pain and did not get an explanation or diagnosis. Conventional X-ray examination may not identify the insufficiency fractures, and some of the patients waited months before an MRI detected the fracture: “*I was sent for a scan, and then they realised what it was*” (patient 3). The missing diagnosis and explanation for the pain led to frustrations: “*No, it’s just that feeling that nobody really believes you*” (patient 1). Some of the patients had a delay of months before the diagnosis was made: “*Sometimes after I’ve been up here, I’ve sat and cried when I got home because I’d got absolutely nothing out of it. After about two weeks, I’d ring up and explain that it hadn’t got any better, and then I’d get called in for the same treatment again*” (patient 8). Most of the patients were treated with intra-articular steroid injection in the fractured joint before the diagnosis was made: “*Then I got an adrenalin injection and it didn’t help really as usual, so I got another one in November and that one didn’t help either*” (patient 3). The diagnoses and explanation of the pain were very important for the patients: “*I’ve never been as happy as when I was told my leg was broken*” (patient 7).

### 3.2. Loss of Functionality

After the diagnosis, the patients were immobilised for several weeks: “*I wasn’t allowed to do anything for two months. I wasn’t even allowed to put any weight on my leg*” (patient 2). Some of the patients were still immobilised during the interview, and they were concerned about whether the fracture would heal as expected: “*I have to wear this boot for the next three months, and if I’m lucky, it’ll be healed after the three months*” (patient 3).

All patients noticed a significant loss of function before the diagnosis was made and during immobilisation: “*I was so unhappy about not being able to do anything … even just getting up at night to go to the toilet*” (patient 7). The patients experienced challenges in performing daily activities: “*I can’t even get to the toilet before I’ve wet myself*” (patient 7). Another patient who was still working said, “*I wasn’t able to work there anymore; I had so much pain. I couldn’t do anything*” (patient 1). Regarding daily activities, the patients mention as their main problem that they were immobilised: “*There were times when I couldn’t even walk!*” (patient 2). Other patients were limited compared to their earlier walking distance: “*Even just being at home, you start thinking, ’How am I going to get to the other end of the house and back?’*” (patient 4). Most of the patients were very active in their daily life before the fracture, and the reduction in walking distance influenced their lives: “*Well, I’ve not been able to do the gardening*” (patient 4). The fear of falling and the fact that the patients experienced a higher risk of falling was also mentioned as another significant burden: “*It’s still the same today … if I don’t look where I’m going, suddenly, whoops, and I am lying on the ground. Just a small twig is enough to make me fall over*” (patient 1). Some of the other patients confirmed this: “S*uddenly, you fall, and you can’t even see any reason for it afterwards*” (patient 5).

These patients face a challenge in having RA and a fracture. During the immobilisation, they were offered crutches, but they were not able to use standard crutches due to their RA: “*I have a problem with using crutches because I don’t have enough strength in my arms to lift myself*” (patient 1). Due to a lack of power in the arms, some of the patients were provided a wheelchair even though it is not optimal due to the physiological consequences of being immobilised: “*I didn’t have enough strength in my hands, so, after the operation, I was in a wheelchair for a while*” (patient 5). A patient asked whether it was possible to get axial crutches. The patients developed ways to solve their problems: “S*o, now I use the office chair in the kitchen when I am cooking*” (patient 4). Another said, “*I couldn’t really do the shopping. I went along sometimes and used the shopping trolley as a walking frame*” (patient 7).

Depending on other people was another psychological factor the patients mentioned: “*I’m lucky enough to have a husband that can cook a little and a grandchild that comes and does the cleaning*” (patient 1). The combination of dependence and the inability of performing daily activities was an issue: “*I used to be able to do the gardening, but now my husband has taken over, it really pains me that I can’t do it*” (patient 4).

Most of the patients who were highly motivated for motion mentioned the importance of being active combined with the challenges of reduced walking distance: “*I wasn’t allowed to do my exercises for my veins and my ankles … When I was in bed, I didn’t have to wear the boot, so I would’ve been able to lift my legs and then do this to stretch them out, but I wasn’t allowed to use my foot*” (patient 9). Another challenge is co-morbidity: “*But then I started getting out of breath and … then they found out that I also have heart fibrillations*” (patient 4). Only one patient was offered rehabilitation: “*The rehabilitation was great…afterwards, I was able to walk properly again*” (patient 3).

Fatigue influenced most of the patients: “*But it was mostly the arthritis and not so much the fracture*” (patient 5), and the other patients confirmed her statement. Mood was also affected for a couple of the patients during the time they were waiting for a diagnosis and during the time they were immobilised: “*Yes, I also get upset and sad about it*” (patient 9). Another patient said, “*I’m the type that tries to hide it and finds it embarrassing*” (patient 10).

Most of the patients experienced more pain than usual with their RA: “*You suddenly have a lot more pain, but you just don´t know the reason*” (patient 5). After treatment with immobilisation, most of the patients had less pain: “*I wasn’t operated (on), and I was allowed to put weight on it. Having the plaster cast on did help with the pain though*” (patient 6).

## 4. Discussion

The present study revealed that patients waited weeks or months to receive a diagnosis, and the patients reported that the waiting time led to uncertainty among them; this reaction is also known among other groups of patients who are misdiagnosed or where the diagnosis is delayed [21,22,23].

Delay in diagnosis is defined as a non-optimal interval of time between onset of symptoms, identification, and initiation of treatment. A delayed diagnosis occurs when the correct diagnosis is delayed due to failure in or untimely ordering of tests (e.g., lab work, colonoscopies, or breast imaging studies) [24]. Patients with RA are accustomed to pain and coping with it, and this is common for patients with RA [25]. This is confirmed in this study where patients do not focus on their pain in general, but on the fact that they experienced a new and inexplicable pain that was more difficult to cope with and which caused uncertainty and worries.

This study confirms that immobilised patients with insufficiency fractures experience functional disability, a decrease in muscle strength, and fatigue [26]. Most of the patients in the present study had an ankle fracture, and a study demonstrated that supervised physical therapy after the immobilisation can reverse the decrease in muscle performance, functional ability, and fatigue [26]. Only the youngest patient was offered rehabilitation, and she was very positive about the effect of the training. It seems very relevant to offer supervised physiotherapy to this group of patients in future practice. This study documents that it is not common practice to offer physiotherapy to this group of patients.

Women with RA demonstrate a twofold increase in osteoporosis [5]. The fact that most of the women in the project were incorrectly treated with glucocorticoids, and afterwards immobilised, further increased the risk of developing osteoporosis [27].

Most patients talked about their risk of falling. It is well known that patients with RA are at increased risk of falling, and when they have fallen once they are more likely to fall again [28,29,30,31]. It is relevant to offer supervised physiotherapy and the aids necessary to prevent falling [28].

RA has an impact on loss of functionality and social life, but the women in this study take pride in their role as a housewife and so forth. More of the women explain that their dependency on other people was very difficult for them to handle. The aids they obtained from the hospital made them more independent, but often the aids were not useful due to their RA. There is a need for special aids for patients with RA. The insufficiency fracture and the immobilisation influenced them negatively, both psychologically and socially. Due to this, it is very important to offer the patients aids that are useful with decreased muscle strength, also documented by Gold et al. [32].

### Limitations and Strengths

A limitation of this study is that therapists conducted the interviews, and this may have influenced the answers, due to the patients’ dependency on the staff of the hospital and thus the unequal balance of power, although there was no ongoing therapist–patient relationship when the interviews were conducted. The interviews took place in the hospital, and, while the particular location was a conference room, this venue may have had an impact on the patients and may have created a medical agenda for the discussion. A limitation of a focus-group interview is that a social control in the group may lead the informants in one direction [33]. Also, several of the patients had fractures years ago, and this led to a risk-of-recall bias [34]. Accordingly, during the interviews, some of the patients were challenged to distinguish between their experiences with the insufficient fracture and other former fractures. Information about comorbidity was not obtained; however, it would have been relevant, as comorbidity can affect the participants’ quality of life.

A strength of this study is that the informants are widely represented in relation to age and activity level. A second strength is the qualitative approach and the use of focus-group interviews to gain an understanding of these patients with RA experiencing an insufficiency fracture, which gives insight into the patients’ universe in relation to their experience of diagnosis, pain, limitations in relation to activities, and offers of rehabilitation and aids. The interactions between participants with diverse characteristics allowed the identification of multiple meanings. Interaction between group participants is considered the distinct advantage of focus-group research, because the group dynamics, agreements, disagreements, and the way people account for their opinions are essential for the content of the data [35].

The knowledge about insufficiency fractures in patients with RA is limited, and there is a need for more focus on this problem to diagnose the insufficiency fracture early by pain onset and to offer the needed rehabilitation and aids.

## 5. Conclusions

In conclusion, the present study documents that patients with RA and insufficiency fractures experience the delayed diagnosis as a burden, and it is stressful for them to wait for an explanation of their pain. Due to the patients’ inability to use crutches or a wheelchair, it is important to find other aids that patients with RA and insufficiency fractures can use. When the patients were immobilised, they experienced the loss of function, but were not offered supervised physical therapy. This points to the need to focus on the diagnosis and guidance of these patients in relation to daily activities, training, and aids.

## Figures and Tables

**Table 1:** Patient characteristics.

	Pseudonym	Gender	Age	RA Duration	Diagnosis Delay
57–69 years (group 1)	1	F	61	38.5 y	1 m 24 d
	2	F	66	12 y	1 m 7 d
	3	F	57	19 y	1 y 9 m 14 d
	4	F	64	12 y	7 m 3 d
	5	F	62	11y	2 m 8 d
	6	F	66	12 y	5 m
71–88 years (group 2)	7	F	77	24 y	6 m 14 d
	8	F	69	32 y	3 m 12 d
	9	F	88	4 y	7 m 3 d
	10	F	69	10 y	6 m 20 d

RA: Rheumatoid arthritis; F: female; y: years; m: months; d: days.
